# Identification and Experimental Validation of LINC00582 Associated with B Cell Immune and Development of Pulpitis: Bioinformatics and In Vitro Analysis

**DOI:** 10.3390/diagnostics13101678

**Published:** 2023-05-09

**Authors:** Wenting Gong, Lilin Hong, Yi Qian

**Affiliations:** 1 Department of Stomatology, The First Affiliated Hospital of Anhui Medical University North District, Hefei 230000, China; 2 Anhui Public Health Clinical Center, Hefei 230000, China

**Keywords:** pulpitis, immune infiltration, long non-coding RNA, human dental pulp cells, LINC00582, B cell

## Abstract

Background: Pulpitis is a common oral disease. Increasing evidence has demonstrated that long non-coding RNAs (lncRNAs) can regulate the immune response in pulpitis. This study focused on finding the key immune-related lncRNAs that regulate the development of pulpitis. Methods: Differentially expressed lncRNAs were analyzed. Enrichment analysis was performed to explore the function of differentially expressed genes. Immune cell infiltration was evaluated with Immune Cell Abundance Identifier. Cell Counting Kit-8 (CCK-8) and lactate dehydrogenase release assays were conducted to measure the viability of human dental pulp cells (HDPCs) and BALL-1 cells. Transwell assay was processed to prove migration and invasion of BALL-1 cells. Results: Our results revealed that 17 lncRNAs were significantly upregulated. Pulpitis-related genes were mainly enriched in inflammatory relative signal pathways. The abundance of various immune cells was significantly abnormal in pulpitis tissues, among which the expression of eight lncRNAs was significantly correlated with the expression of B cell marker protein CD79B. As the most relevant lncRNA for B cells, LINC00582 could regulate the proliferation, migration, invasion, and CD79B expression of BALL-1 cells. Conclusions: Our study identified eight B cell immune-related lncRNAs. Meanwhile, LINC00582 has a positive effect on B cell immunity in the development of pulpitis.

## 1. Introduction

Pulpitis is a pulp disease caused by bacterial infection, mechanical stimulation, and chemical stimulation. Pulpitis can lead to a change in the pulp blood flow system, which can induce vasodilation, increase vascular permeability near the pulp, and increase intramedullary pressure [1,2]. Usually, pulpitis causes irreversible damage to the pulp. This is mainly because the occurrence of pulpitis is often accompanied by the loss of the pulp regeneration ability of organisms [3,4]. Pulpitis is a common disease in oral clinics. At present, root canal treatment is the main treatment for pulpitis [5]. Although root canal treatment can effectively relieve the pain of dental pulp and inhibit the further development of inflammation, it can also seriously damage the activity of dental pulp tissue in the process of operation [5,6]. After treatment, the pulp tissue of the patient is completely cut off from the nutritional supply to the dental enamel tissue, greatly reducing the service life of the tooth. If the treatment is not effective, pulpitis can further progress to pulp necrosis, periapical periodontitis, and even serious oral infection [7]. Therefore, it is of great significance to clarify the molecular mechanism of pulpitis and find a way to treat pulpitis without destroying the activity of pulp tissue.

Pulpitis is a complex pathological lesion caused by the interaction of multiple factors. For stem cell differentiation, stem cells are involved in inflammation that occurs during restorative dentin formation in pulpitis [8]. Dental pulp stem cell (DPSC)-based odontogenic regeneration in inflammatory conditions is also important in the process of pulpitis [3]. In addition, as an important subtype of mesenchymal stem cells, stem cells from human exfoliated deciduous teeth (SHEDs) can also affect the progression of pulpitis by regulating cell differentiation [9]. However, the pathogenesis of pulpitis is not fully understood. In the exploration of the pathogenesis of pulpitis, the immune response is considered one of the important pathogenesis factors by some researchers [10,11]. Some reports have shown that many cytokines, such as tumor necrosis factor-α (TNF-α) or interleukin (IL), could release in the dental pulp when it is infected or stimulated to induce inflammation [12,13]. TNF-α can promote the release of neutrophil inflammatory cytokines and the activation of natural killer (NK) cells. These biological processes can promote the inflammatory effect of pulpitis [14]. ILs in immune cells can also play an immunomodulatory role by activating immune cells and promoting the expression of cytokines [11,15]. Therefore, further exploring the immune infiltration of immune cells in the process of pulpitis and finding the key immune cells in the regulation of pulpitis can provide a theoretical basis for the treatment of pulpitis.

In recent years, numerous studies have shown that abnormal expression of non-coding RNAs can lead to a variety of different diseases, including inflammatory diseases, neurological disorders, and cancer. A variety of non-coding RNAs, including long non-coding RNAs (lncRNAs), have also been found to play a regulatory role in oral-related diseases. Previous reports have found that some lncRNAs were differentially expressed between the oral tissues of healthy people and oral tissues of pulpitis patients [16,17]. These lncRNAs may affect the occurrence and progression of pulpitis. lncRNA maternally expressed gene 3 (MEG3) has been proven to have a negative effect on inflammation and regeneration of the dentin-pulp complex in pulpitis [18]. Some researchers also revealed that the knockdown of plasmacytoma variant translocation 1 (PVT1) might suppress the damage in pulpitis cell models induced by lipopolysaccharide (LPS) [19]. In addition, NUT family member 2A-AS1 (NUTM2A-AS1) has also been regarded as a key factor in regulating apoptosis and inflammation in dental pulp cells [20]. However, no reports focus on how lncRNA regulates the infiltration level of related immune cells in the development of pulpitis. 

In the present study, key immune cells and immune response signaling pathways associated with the development of pulpitis were identified with bioinformatic techniques. In pulpitis, we also found immune-related lncRNAs that could bind to CD79B and regulate B cell infiltration. These findings showed that some lncRNAs were required for regulating immune infiltration in pulpitis. 

## 2. Materials and Methods

### 2.1. Data Analysis

Raw data were downloaded from GSE92681 in Gene Expression Omnibus (GEO) database (https://www.ncbi.nlm.nih.gov/geo/query/acc.cgi?acc=GSE92681, accessed on 7 September 2022) and normalized with the quantile algorithm using Genespring software (version 14.8, Agilent Technologies, Palo Alto, CA, USA). Patients were diagnosed with irreversible pulpitis in accordance with the endodontics diagnoses system from the American Association of Endodontists. Table 1 provides specific information about the samples. Uniform Manifold Approximation and Projection (UMAP) analysis was used to validate repeatability of sample. The samples with poor repeatability were removed (GSM2434473 and GSM2434475). Differentially expressed genes were analyzed by using the limma package in R (*p* value < 0.05, |logFC| > 1). Volcano plot and heatmap were generated using R tool to show differentially expressed genes explicitly. The z-score for each gene was calculated by subtracting the mean level from the raw expression level, then dividing the difference by the standard deviation. Benjamini-Hochberg adjustment for *p* values was applied to the limma analysis. 

### 2.2. Functional Enrichment Analysis

Functional annotation of differentially expressed genes between normal and inflamed pulp tissue was processed using R package “clusterprofiler”; Gene Ontology (GO) and Kyoto encyclopedia of genes and genomes (KEGG) enriched pathways were visualized by using bar plot and scatter plot. Expression data and metadata information were enrolled and processed using R package “clusterprofiler” in gene set enrichment analysis (GSEA). GSEA plot functions were then used to visualize GSEA enrichment results. The pathways with enrichment significance *p* value < 0.05 were considered as pathways activity significantly changed between normal and inflamed pulp tissue.

### 2.3. Immune Cell Infiltration Analysis

The normalized gene expression data were uploaded to the Immune Cell Abundance Identifier (ImmuCellAI) (http://bioinfo.life.hust.edu.cn/web/ImmuCellAI/ accessed on 24 September 2022). ImmuCellAI is a website that can use a gene set signature-based method to precisely estimate the infiltration score of 24 immune cell types, including 18 T-cell subsets, and to predict the immune cell infiltration status.

### 2.4. Correlation Analysis between LncRNA and mRNA

The correlation analysis was calculated by the R package called “corrplot” by using the expression levels of the selected lncRNAs and mRNAs.

### 2.5. Tissue Samples

To further verify the expression of the differentially expressed lncRNAs, 6 human inflamed pulp tissue samples and 6 normal pulp tissue samples were collected according to reports from previous studies [16,17,18]. Pulp tissue samples were obtained from the First Affiliated Hospital of Anhui Medical University. Normal pulp tissues were collected from healthy third molars or teeth extracted for orthodontic purposes. Inflamed pulp tissues were extracted from teeth diagnosed with irreversible pulpitis in accordance with the endodontics diagnoses system from the American Association of Endodontists. Any teeth that had periodontitis were excluded. Patients who had a compromised immune system or those who were taking medications known to influence the immune response were excluded from the study. All samples were immediately placed in liquid nitrogen and subsequently frozen at −80 °C. The study was approved by the Ethical Committee of Anhui Public Health Clinical Center (PJ-YX2023-005).

### 2.6. Cell Culture and Transfection

Human dental pulp cells (HDPCs) and BALL-1 cells were purchased from American Tissue Culture Collection (ATCC, Manassas, VA, USA). Cells were added into Dulbecco’s modified Eagle’s medium (DMEM) (Gibco, New York, NY, USA). The medium including 100 units/mL penicillin/streptomycin and 10% fetal bovine serum (FBS, Gibco, New York, NY, USA). Cell culture condition was a 37 °C incubator in a humidified 5% CO_2_. The overexpression of negative controls (oe-NC) and oe-LINC00582 were obtained from GenePharma (Shanghai, China). The cells were transfected using Lipofectamine 2000™ (Invitrogen, Thermo Fisher Scientific, Waltham, MA, USA) following the manufacturer’s suggestions. LPS was purchased from Solarbio (Beijing, China). DMEM was used to dissolve LPS. 50 ng/mL LPS was used to treated HDPCs for 24 h. The control group was HDPCs treated with equivalent DMEM.

### 2.7. Real-Time Quantitative Polymerase Chain Reaction (RT-qPCR)

Total RNA was separated with TRIzol reagent (Invitrogen, Thermo Fisher Scientific, Waltham, MA, USA). To verify expression results, 2 μg of total RNA was used for cDNA synthesis with a Takara PrimeScript RT Reagent kit (Takara, Shiga, Japan). A housekeeping glyceraldehyde 3-phosphate dehydrogenase (GAPDH) gene was used as an endogenous control. The cDNA was used for RT-qPCR with SYBR Green Master Mix (Takara, Shiga, Japan) on an ABI PRISM 7500 RT-qPCR System (Applied Biosystems, Thermo Fisher Scientific, Waltham, MA, USA). Primers were list in Supplementary Table S1. The relative expression of these RNAs was analyzed with the 2^−ΔΔCt^ method.

### 2.8. Cell Counting Kit-8 (CCK-8) Assay

CCK-8 solution (ab228554, Abcam, Burlingame, CA, USA) was recommended to assess cell viability of HDPCs and BALL-1 cells. The cells were inoculated into 96-well plates and then cultured in an incubator with 5% CO_2_ at 37 °C for 2 h. Add 10 μL CCK-8 reagent to the well of each group after time and mix, then incubate in an incubator for 2 h. The optical density (OD) at 450 nm was measured with a microplate reader.

### 2.9. Lactate Dehydrogenase (LDH) Release Detection Assay

4 × 10^4^ cells HDPCs were seeded into 24-well plates. LDH was used to specific transfection. LDH activity was measured with a commercially available assay kit (C20300, Thermo Fisher Scientific, Waltham, MA, USA). After adding stop buffer, we measured OD490 value. Percent cytotoxicity = 100 × Experimental LDH Release (OD490)/Maximum LDH Release (OD490).

### 2.10. Transwell Assay of BALL-1 Cells

BALL-1 cells were cultured at 4 × 10^4^ cells/mL. The cells were added into top of Transwell chamber (Corning, New York, NY, USA) including medium containing 0.1% BSA (Solarbio, Beijing, China). The medium containing 10% FBS was added into lower chamber. After the cells were cultured for 24 h, the cell number in the lower chamber was counted using a hemocytometer. For invasion assay, a Matrigel (Becton Dickinson, Franklin Lake, NJ, USA) was thawed at 4 °C overnight before being placed into the upper chamber and incubated at 37 °C for 1 h.

### 2.11. Transwell Assay of BALL-1 Cells

One-way ANOVA followed by Tukey’s post hoc test was used for the comparisons of multiple groups, while Student’s *t*-test was employed for comparisons between two groups. *p* value less than 0.05 represents significant difference.

## 3. Results

### 3.1. Identification and Characteristics of LncRNAs between Normal and Pulpitis Tissue

**Figure 1:**
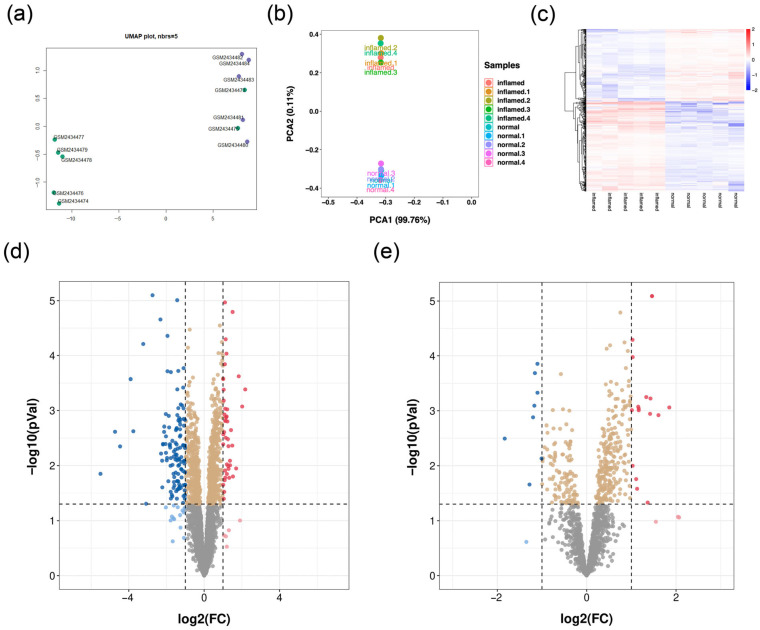
Overview of mRNA and lncRNA levels. (**a**) Uniform Manifold Approximation and Projection (UMAP) analysis was used to validate repeatability of sample. (**b**) Principal Component Analysis (PCA) showed good biological replicant. (**c**) Heatmap showed 163 genes were differentially expressed between normal tissues and pulpitis tissues. (**d**) Volcano plot showed 120 genes (blue) were downregulated in pulpitis tissues, and 43 genes (red) were upregulated in pulpitis tissues. (**e**) Volcano plot showed 8 genes (blue) were downregulated in pulpitis tissues, and 17 genes (red) were upregulated in pulpitis tissues.

To screen the differentially expressed genes and lncRNAs in normal tissues and pulpitis tissues, the microarray data were downloaded from GEO, and then the differentially expressed genes were calculated. Firstly, UMAP and Principal Component Analysis (PCA) were used to verify sample repeatability and exclude low-quality samples (Figure 1a,b). Furthermore, the results of differentially expressed genes and differentially expressed lncRNAs were analyzed. In total, 43 genes were upregulated, and 120 genes were downregulated in pulpitis tissues (Figure 1c,d). As shown in Figure 1e, we observed seventeen lncRNAs were significantly upregulated, and eight lncRNAs were significantly downregulated in pulpitis tissues. These differentially expressed genes and lncRNAs were considered candidate genes for further analysis.

### 3.2. Analysis of LncRNAs Differentially Abundant in Normal Pulp Tissues Compared with Inflamed Pulp Tissues

**Figure 2:**
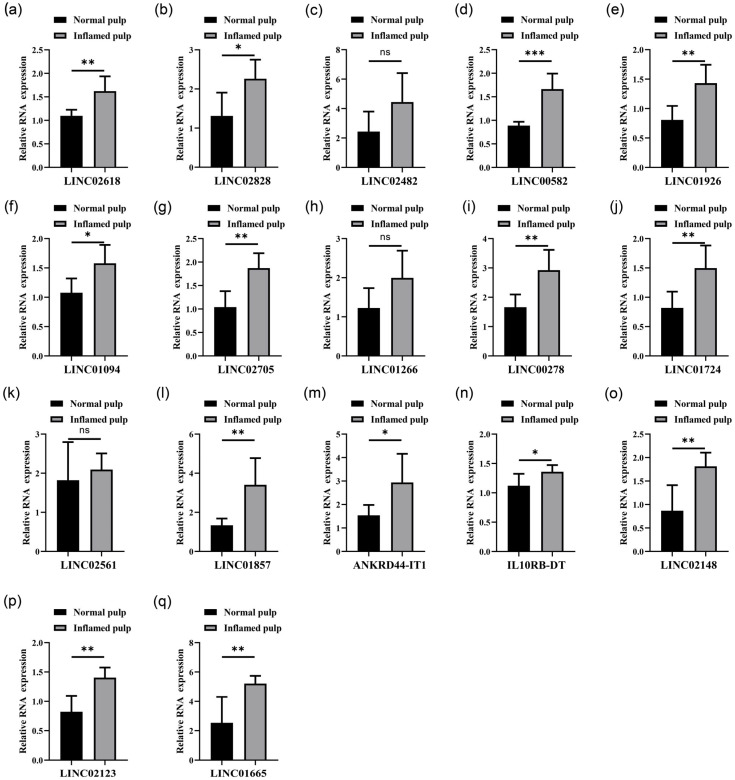
The expression levels of 17 lncRNAs in normal pulp tissues and inflamed pulp tissues. (**a**–**q**) The expression levels of 17 lncRNAs in normal pulp tissues and inflamed pulp tissues were measured with Real-time quantitative polymerase chain reaction (RT-qPCR). Data are shown as the mean ± SD. * p < 0.05, ** p < 0.01, *** p < 0.001, ns represents *p* > 0.05.

To verify the expression of the 17 lncRNAs, RT-qPCR was performed. The results showed that 14 lncRNAs were significantly highly expressed in inflamed pulp tissues (Figure 2). Among them, 10 lncRNAs showed higher expression level in inflamed pulp tissues (Figure 2). Hence, these 10 lncRNAs were taken as the target lncRNAs for further study.

### 3.3. Functional Annotation of Differentially Expressed Genes between Normal and Pulpitis Tissue 

**Figure 3:**
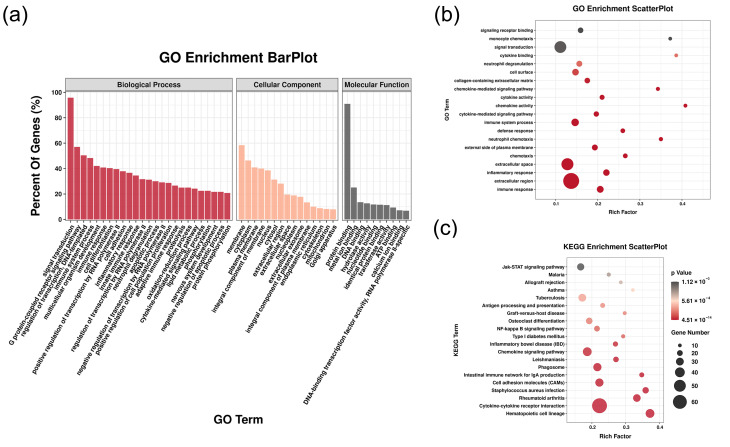
The enrichment analysis of different expressed genes. (**a**) Bar-plot of Gene Ontology (GO) enrichment analysis. (**b**) Scatter-plot of GO enrichment analysis. (**c**) Scatter-plot of Kyoto encyclopedia of genes and genomes (KEGG) enrichment analysis.

**Figure 4:**
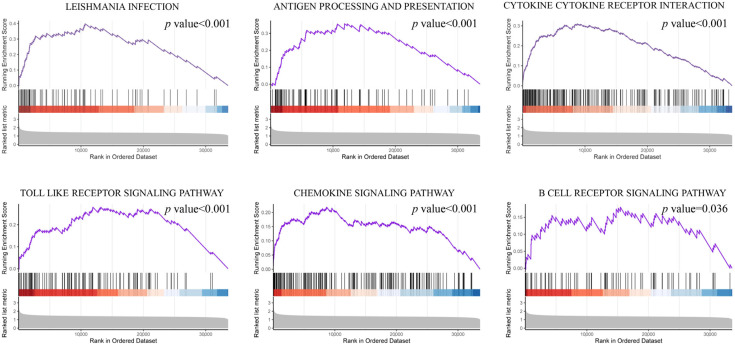
Gene set enrichment analysis (GSEA) of differentially expressed genes between normal and pulpitis tissue.

As shown in GO results, biological process ontology, immune system process, immune response, and inflammatory response were the top affected pathways (Figure 3a). Similar results were also shown in the GO enrichment scatterplot (Figure 3b). The inflammatory relative signal pathways, such as monocyte chemotaxis, cytokine binding, and neutrophil degranulation, were listed as top pathways judged by the *p* value. According to KEGG pathway analysis, differentially expressed genes were found to be enriched in several key pathways in the inflammatory relative signal pathway, such as antigen processing and presentation and the chemokine signaling pathway (Figure 3c). For GSEA, we observed that a lot of inflammation-relative pathways were significantly enriched (Figure 4). Further analysis of GSEA results indicated that among these inflammatory relative pathways, the B cell relative pathway was one of the most affected pathways (Figure 4). The results suggested that B cells might play an important role in the development of pulpitis tissue.

### 3.4. Immune Cell Abundance Identification between Normal and Inflamed Tissue

**Figure 5:**
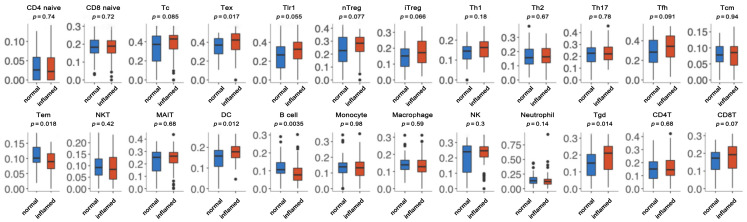
Infiltration abundance of 24 types of immune cells in normal group and inflamed group.

To further investigate whether B cells mediate pulpitis, ImmuCellAI was used to calculate the immune infiltration score. As shown in Figure 5, exhausted CD8 T (Tex) cells, dendritic cells (DC), and Tgd cells were significantly highly abundant in inflamed tissue. Furthermore, Tem cells and B cells were also downregulated in the inflamed tissue. Among them, the abundance of B cells was most significantly different between normal and inflamed tissue, which is as expected from the GSEA results.

### 3.5. Correlation Analysis between LncRNAs and B Cell Activate Marker CD79B

**Figure 6:**
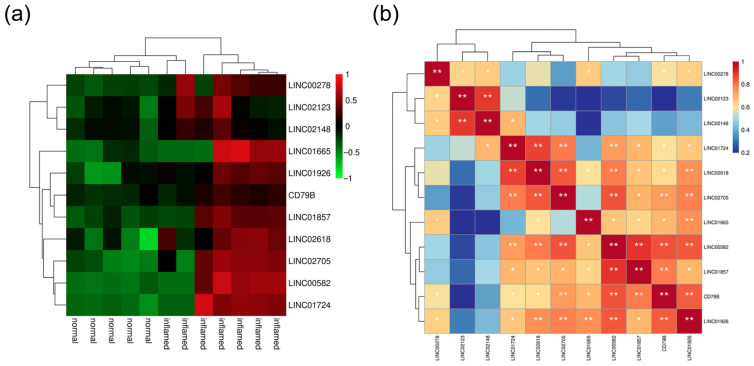
Correlation analysis between lncRNAs and B cell activate marker CD79B. (**a**) Heatmap showed the top 10 differentially expressed lncRNAs. (**b**) Predicted binding sites between CD79B and LINC00582 with RIsearch. * p < 0.05, ** p < 0.01.

In the following study, we further explored lncRNAs that affect B cell infiltration in dental pulp by regulating mRNA transcription. As a key protein regulating the B-cell receptor signaling pathway, CD79B was also used to analyze the correlation between CD79B and 10 differentially expressed lncRNAs (Figure 6a). As shown in Figure 6b, eight lncRNAs have a strong connection to CD79B with a significant *p*-value, especially LINC00582 (correlation: 0.903).

### 3.6. Overexpression of LINC00582 Could Not Regulate Biological Function of HDPCs

**Figure 7:**
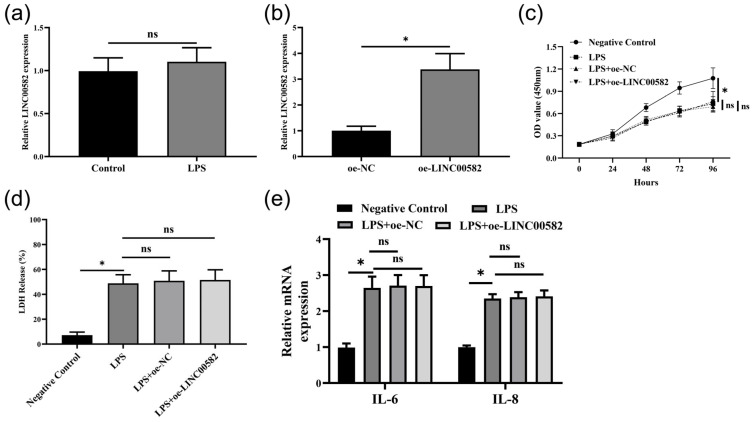
Overexpression of LINC00582 could not regulate biological function of HDPCs. (**a**) The expression level of LINC00582 in HPDCs and lipopolysaccharide (LPS)-induced HPDCs. (**b**) The expression level of LINC00582 in HPDCs of overexpression of negative controls (oe-NC) and oe-LINC00582. (**c**) Cell viability in Negative Control, LPS, LPS+oe-NC, and LPS+oe-LINC00582 group with Cell Counting Kit-8 (CCK-8) assay. (**d**) The cytotoxicity of HPDCs was detected by LDH release detection assay. (**e**) The expression levels of IL-6 and IL-8 in Negative Control, LPS, LPS+oe-NC, and LPS+oe-LINC00582 group. Data are shown as the mean ± SD. * p < 0.05, ns represents *p* > 0.05.

To test whether LINC00582 was involved in the effect of dental pulp cells, the HPDCs were treated with LPS, and the overexpressing LINC00582 cell line was successfully constructed (Figure 7a,b). The viability of HPDCs was measured with CCK-8. LPS could inhibit the proliferation ability of HPDCs (Figure 7c). However, LINC00582 could not regulate the viability of HPDCs. The same trend was also shown in LDH release and the expression of inflammatory cytokines (IL-6 and IL-8) (Figure 7d,e). These findings revealed that high expression of LINC00582 could not regulate the biological function of HDPCs.

### 3.7. Overexpression of LINC00582 Promoted Proliferation, Migration, Invasion, and the Expression of CD79B in BALL-1 Cells

**Figure 8:**
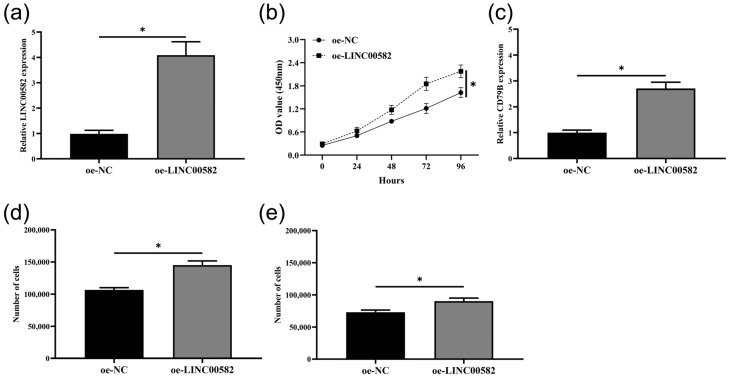
LINC00582 promoted proliferation, migration, invasion, and the expression of CD79B in BALL-1 cells. (**a**) The expression level of LINC00582 in BALL-1 cells of overexpression of negative controls (oe-NC) and oe-LINC00582. (**b**) Cell viability in oe-NC and oe-LINC00582 group with Cell Counting Kit-8 (CCK-8) assay. (**c**) The expression level of CD79B in BALL-1 cells of oe-NC and oe-LINC00582. (**d**) Migration ability in oe-NC and oe-LINC00582 group with Transwell assay. (**e**) Invasion ability in oe-NC and oe-LINC00582 group with Transwell assay. Data are shown as the mean ± SD. * p < 0.05.

To further verify the regulatory effect of LINC00582 on B cells, BALL-1 cells were selected for subsequent experiments (Figure 8a). After that, our experimental results showed that LINC00582 could promote the proliferation of BALL-1 cells (Figure 8b). Moreover, overexpression of LINC00582 also increased the expression of CD79B in BALL-1 cells (Figure 8c). The results of the transwell assay showed that the overexpression of LINC00582 could upregulate migration and invasion numbers of BALL-1 cells. (Figure 8d,e). These results suggested that LINC00582 might indirectly regulate the development of pulpitis by regulating the expression of CD79B and affecting the activity of B cells.

## 4. Discussion

Pulpitis is an inflammatory disease that affects the pulp tissue. External stimuli such as bacterial infection or mechanical damage may cause the occurrence of this disease [21]. During the development of pulpitis, cells, including HPDCs, immune cells, and endothelial cells, secrete several inflammatory factors, such as ILs and chemokines [13,22]. These inflammatory biomarkers play a significant role in regulating the development of pulpitis. With the attention of clinical researchers on pulpitis, the regulatory mechanisms and influencing pathways of some inflammatory factors in pulp-related cells have been gradually reported. CXC-chemokine receptor 4 (CXCR4) combined with recombinant platelet/endothelial cell adhesion molecule (PECAM1) could regulate inflammatory cell infiltration by activating the Nuclear Factor-kappa B (NF-κB) signaling pathway in pulpitis [23]. IL-17 has also been regarded as an inflammatory mediator in dental pulp inflammation. The expression level of IL-17 could be regulated by mitogen-activated protein kinase (MAPK)-p38/externally regulated kinase (ERK) pathway [24]. Although current studies have indicated the relationships between pulpitis and some cell factors, there are still many genes that are abnormally expressed in pulpitis, and the relevant pathways that regulate the development of pulpitis cannot be identified. In the present study, differentially expressed genes were analyzed in pulp tissues from pulpitis patients and normal participants. Enrichment analysis was also used to explore the possible functions and pathways affected by these differential genes.

The inflammatory reaction-related pathway is always one of the most important pathways in the development of pulpitis. Many researchers have also found that the genes associated with pulpitis were generally enriched in immune or inflammatory pathways through a variety of enrichment analysis methods. Xin and his partners discovered 843 differentially expressed genes related to pulpitis. They found these genes mainly enriched in chemokine, TNF, and IL-17 signaling pathways, which belonged to inflammatory reaction-related pathways [25]. A total of 470 differentially expressed genes related to pulpitis were found in the research by Chen and his team. These genes were also associated with cytokine-cytokine receptor interaction, chemokine signalling pathway, and NF-κB signalling pathway [26]. The enrichment results of this study also showed that the differential genes of pulpitis were generally involved in inflammatory processes. This result further clarified the importance of inflammatory response in the development of pulpitis. Except this, GSEA results indicated that among these inflammatory relative pathways, the B cell relative pathway was one of the most affected pathways, such as the FC gamma mediated phagocytosis pathway and the B cell receptor signaling pathway. The results suggested infiltration of immune cells was closely related to pulpitis.

Dental pulp stimulates the innate immune system, which may aid in the defense against bacterial invasion. In this process, a variety of immune cells play an important role. Therefore, exploring the difference in immune cell infiltration between pulpitis tissues and non-pulpitis tissues is helpful to find the key immune cells involved in the development of pulpitis. The results of our research showed that Tex cells, dendritic cells (DC), and Tgd cells were significantly highly abundant in inflamed tissue. Furthermore, Tem cells and B cells were also downregulated in the inflamed tissue. In past studies, the quantity of DCs was found significantly upregulated with experimentally induced pulpitis [11]. In whole murine tooth, CD11c+DCs with processes in the dentinal tubule were significantly increased in the dental pulpitis model induced by mechanical and chemical irritation [27]. In the present study, the abundance of B cells was most significantly different between normal and inflamed tissues. B cell receptors or related pathways have been found to be associated with inflammatory infiltration in pulpitis [23,28]. Some researchers have found that a few B cells could appear in early pulpitis. CD4^+^T cells could promote the number of B cells via binding specific immunoglobulin on B cells in pulpitis [29]. Both T cytotoxic-1 and T cytotoxic-2 cells could also induce strong inflammatory reactions to stimulate the proliferation and differentiation of B cells [30,31]. B cells play an important role in the inflammatory response of pulp tissue. Some studies suggest that the proliferation and differentiation of B cells were an indication of homeostasis or chronicity of the disease state [32]. The significantly higher numbers of B cells and plasma cells found in irreversible pulpitis samples also supported a type-2 immune response beneath deep caries [33,34]. In addition, B cells can also function as antigen-presenting cells (APCs), modulate DCs functions, and produce cytokines such as IL-10, IL-4, and IFN-γ in response to pathogens [35,36]. Earlier reports also found that inflamed pulps contained higher levels of B-cell marker proteins than noninflamed pulp [37,38]. On the other hand, studies on B-cell regulation of pulpitis progression are still scarce. Therefore, it is of certain significance to explore the infiltration of B cells in the process of pulpitis for finding a new mechanism of pulpitis.

Non-coding RNA is a key factor in the regulation of pulpitis response. With the popularization of high-throughput RNA sequencing technology, a great deal of differentially expressed non-coding RNAs, including lncRNAs, have also been demonstrated in pulpitis tissues [17]. In this study, we also screened 25 differentially expressed lncRNAs. The top 10 significantly changed lncRNAs were regarded as key lncRNAs in the present study. Most lncRNAs belonging to these 10 lncRNAs were found to be associated with pulpitis for the first time in this study. LINC00278 has been reported to be closely associated with the development of a variety of squamous cell carcinomas [39,40]. LINC00582 is also abnormally expressed in the tissues and blood of patients with some diseases [41,42]. For LINC01857, past studies have shown that the lncRNA could regulate the biological activity of various cancer cells’ competitive endogenous RNA (ceRNA) activity [43,44,45]. Moreover, the diagnosis ability of LINC01857 to pulpitis has also been verified [46]. Hence, the exploration of the association between these lncRNAs and pulpitis was of great value for discovering novel diagnostic markers and therapeutic targets.

In this study, we focused on the role of B cells in the regulation of pulpitis. In the following study, we further explored lncRNAs that affected B cell infiltration in dental pulp by regulating mRNA transcription. In this study, LINC00582 was found to be most related to the B cell marker protein CD79B. In previous studies, LINC00582 has been found that it was associated with the outcome of oral cancer [41]. We also first explored whether LINC00582 could directly regulate dental pulp cells. The results showed that HPDCs were not regulated by LINC00582 in terms of cell proliferation, cytotoxicity, or inflammatory response. Therefore, we hypothesized that the effect of LINC00582 on pulpitis was indirectly caused by regulating the activity of B cells. Subsequent results also proved that overexpression of LINC00582 could regulate the proliferation, migration, invasion, and CD79B expression of BALL-1 cells. These results and reports suggest that LINC00582 may regulate B-cell infiltration and influences the inflammatory response in pulpitis.

While the association between LINC00582 and pulpitis has been verified, several limitations remain. First, although we systematically searched the open-source database, the total number of pulp samples is still scarce. As a result, alpha and beta errors may affect predictive accuracy. Second, more clinical information is still needed to further analyze correlations between molecular findings and clinical characteristics/prognosis for patients, providing a more intuitive explanation for our results. Furthermore, differential expression of lncRNAs has been found in pulpitis tissues, but due to the small number of samples, it is necessary to expand the number of samples for further verification in future studies. Finally, the results of this study cannot explain how an altered expression of LINC00582 in the inflamed dental pulp can be of clinical utility in the treatment and prevention of pulpitis. The molecular mechanism of how LINC00582 regulates the action of B cells on pulp cells has not been fully elucidated. Therefore, more in vivo and in vitro experiments are needed for future research. 

## 5. Conclusions

Generally, we used bioinformatics methods to explore the inflammatory pathways in pulpitis. The results proved the infiltration levels of various immune cells in pulpitis tissues were significantly different from those in normal pulp tissues. We revealed that the expression of eight lncRNAs was significantly correlated with the expression of B cell marker protein CD79B. B cells were regarded as important immune cells, and LINC00582 could promote the biological activity of B cells in our research. These findings provide new insights into the pathogenesis of pulpitis and may identify potential diagnostic and therapeutic strategies for future studies.

## Figures and Tables

**Table 1:** The baseline information of GSE92681.

Samples	Age (Years)	Diagnostic Information	Sample Information
Pulpitis tissue 1	26	Irreversible pulpitis	Inflamed pulp tissue
Pulpitis tissue 2	27	Irreversible pulpitis	Inflamed pulp tissue
Pulpitis tissue 3	29	Irreversible pulpitis	Inflamed pulp tissue
Pulpitis tissue 4	25	Irreversible pulpitis	Inflamed pulp tissue
Pulpitis tissue 5	27	Irreversible pulpitis	Inflamed pulp tissue
Pulpitis tissue 6	29	Irreversible pulpitis	Inflamed pulp tissue
Pulpitis tissue 7	24	Irreversible pulpitis	Inflamed pulp tissue
Normal pulp tissue 1	25	Healthy pulp tissue	Third molars extracted for orthodontic purposes
Normal pulp tissue 2	24	Healthy pulp tissue	Third molars extracted for orthodontic purposes
Normal pulp tissue 3	22	Healthy pulp tissue	Third molars extracted for orthodontic purposes
Normal pulp tissue 4	24	Healthy pulp tissue	Third molars extracted for orthodontic purposes
Normal pulp tissue 5	25	Healthy pulp tissue	Third molars extracted for orthodontic purposes

## Data Availability

The data that support the findings of this study are available in GEO at https://www.ncbi.nlm.nih.gov/geo/query/acc.cgi?acc=GSE92681, reference number GSE92681, accessed on 7 September 2022. The datasets used and/or analyzed during the current study are available from the corresponding author on reasonable request.

## Supplementary Materials

The following supporting information can be downloaded at: https://www.mdpi.com/article/10.3390/diagnostics13101678/s1, Table S1: The primers used in this study.

## References

[1] Leng S., Liu L., Xu W., Yang F., Du J., Ye L., Huang D., Zhang L. (2022). Inflammation down regulates stromal cell-derived factor 1alpha in the early phase of pulpitis. Cytokine.

[2] Gufran K., Mirza M.B., Robaian A., Alqahtani A.S., Alqhtani N.R., Alasqah M., Alsakr A.M. (2022). A Prospective Clinical Study Evaluating the Efficacy of Intra-Ligamentary Anesthetic Solutions in Mandibular Molars Diagnosed as Symptomatic Irreversible Pulpitis with Symptomatic Apical Periodontitis. Healthcare.

[3] Zhong T.Y., Zhang Z.C., Gao Y.N., Lu Z., Qiao H., Zhou H., Liu Y. (2019). Loss of Wnt4 expression inhibits the odontogenic potential of dental pulp stem cells through JNK signaling in pulpitis. Am. J. Transl. Res..

[4] Yan H., Oshima M., Raju R., Raman S., Sekine K., Waskitho A., Inoue M., Inoue M., Baba O., Morita T. (2021). Dentin-Pulp Complex Tissue Regeneration via Three-Dimensional Cell Sheet Layering. Tissue Eng. Part C Methods.

[5] Lu Y., Liu Z., Huang J., Liu C. (2020). Therapeutic effect of one-time root canal treatment for irreversible pulpitis. J. Int. Med. Res..

[6] Bamini L., Anand Sherwood I., Abbott P.V., Uthandakalaipandian R., Velu V. (2020). Influence of anti-inflammatory irrigant on substance P expression for single-visit root canal treatment of teeth with irreversible pulpitis. Aust. Endod. J..

[7] Bertossi D., Barone A., Iurlaro A., Marconcini S., De Santis D., Finotti M., Procacci P. (2017). Odontogenic Orofacial Infections. J. Craniofac. Surg..

[8] Ustiashvili M., Kordzaia D., Mamaladze M., Jangavadze M., Sanodze L. (2014). Investigation of functional activity human dental pulp stem cells at acute and chronic pulpitis. Georgian Med. News.

[9] Bhandi S., Alkahtani A., Mashyakhy M., Abumelha A.S., Albar N.H.M., Renugalakshmi A., Alkahtany M.F., Robaian A., Almeslet A.S., Patil V.R. (2021). Effect of Ascorbic Acid on Differentiation, Secretome and Stemness of Stem Cells from Human Exfoliated Deciduous Tooth (SHEDs). J. Pers. Med..

[10] Galler K.M., Weber M., Korkmaz Y., Widbiller M., Feuerer M. (2021). Inflammatory Response Mechanisms of the Dentine-Pulp Complex and the Periapical Tissues. Int. J. Mol. Sci..

[11] Renard E., Gaudin A., Bienvenu G., Amiaud J., Farges J.C., Cuturi M.C., Moreau A., Alliot-Licht B. (2016). Immune Cells and Molecular Networks in Experimentally Induced Pulpitis. J. Dent. Res..

[12] Ghattas Ayoub C., Aminoshariae A., Bakkar M., Ghosh S., Bonfield T., Demko C., Montagnese T.A., Mickel A.K. (2017). Comparison of IL-1beta, TNF-alpha, hBD-2, and hBD-3 Expression in the Dental Pulp of Smokers Versus Nonsmokers. J. Endod..

[13] Kermeoglu F., Aksoy U., Sebai A., Savtekin G., Ozkayalar H., Sayiner S., Sehirli A.O. (2021). Anti-Inflammatory Effects of Melatonin and 5-Methoxytryptophol on Lipopolysaccharide-Induced Acute Pulpitis in Rats. Biomed. Res. Int..

[14] Thanapati S., Ganu M., Giri P., Kulkarni S., Sharma M., Babar P., Ganu A., Tripathy A.S. (2017). Impaired NK cell functionality and increased TNF-alpha production as biomarkers of chronic chikungunya arthritis and rheumatoid arthritis. Hum. Immunol..

[15] Shindo S., Kumagai T., Shirawachi S., Takeda K., Shiba H. (2021). Semaphorin3A released from human dental pulp cells inhibits the increase in interleukin-6 and CXC chemokine ligand 10 production induced by tumor necrosis factor-alpha through suppression of nuclear factor-kappaB activation. Cell Biol. Int..

[16] Lei F., Zhang H., Xie X. (2019). Comprehensive analysis of an lncRNA-miRNA-mRNA competing endogenous RNA network in pulpitis. PeerJ.

[17] Du J., Liu L., Yang F., Leng S., Zhang L., Huang D. (2022). Identification of Immune-Related lncRNA Regulatory Network in Pulpitis. Dis. Markers.

[18] Liu M., Chen L., Wu J., Lin Z., Huang S. (2021). Long noncoding RNA MEG3 expressed in human dental pulp regulates LPS-Induced inflammation and odontogenic differentiation in pulpitis. Exp. Cell Res..

[19] Xia L., Wang J., Qi Y., Fei Y., Wang D. (2022). Long Non-coding RNA PVT1 is Involved in the Pathological Mechanism of Pulpitis by Regulating miR-128-3p. Oral. Health Prev. Dent..

[20] Wang X., Sun H., Hu Z., Mei P., Wu Y., Zhu M. (2021). NUTM2A-AS1 silencing alleviates LPS-induced apoptosis and inflammation in dental pulp cells through targeting let-7c-5p/HMGB1 axis. Int. Immunopharmacol..

[21] Van de Vijfeijken S., Zigterman B.G.R., Helmers R., Dubois L. (2022). The dentogenic inflammation, diagnosis, course and treatment. Ned. Tijdschr. Tandheelkd..

[22] Yong D., Cathro P. (2021). Conservative pulp therapy in the management of reversible and irreversible pulpitis. Aust. Dent. J..

[23] Liu Y., Zhang Z., Li W., Tian S. (2020). PECAM1 Combines With CXCR4 to Trigger Inflammatory Cell Infiltration and Pulpitis Progression Through Activating the NF-kappaB Signaling Pathway. Front. Cell Dev. Biol..

[24] Liu M., Zhao Y., Wang C., Luo H., A P., Ye L. (2019). Interleukin-17 plays a role in pulp inflammation partly by WNT5A protein induction. Arch. Oral. Biol..

[25] Xin B., Lin Y., Tian H., Song J., Zhang L., Lv J. (2021). Identification of Pulpitis-Related Potential Biomarkers Using Bioinformatics Approach. Comput. Math. Methods Med..

[26] Chen M., Zeng J., Yang Y., Wu B. (2020). Diagnostic biomarker candidates for pulpitis revealed by bioinformatics analysis of merged microarray gene expression datasets. BMC Oral. Health.

[27] Hong S., Park Y.H., Lee J., Moon J., Kong E., Jeon J., Park J.C., Kim H.R., Kim P. (2021). 3D Visualization of Dynamic Cellular Reaction of Pulpal CD11c+ Dendritic Cells against Pulpitis in Whole Murine Tooth. Int. J. Mol. Sci..

[28] Hahn C.L., Liewehr F.R. (2007). Update on the adaptive immune responses of the dental pulp. J. Endod..

[29] Lanzavecchia A. (1990). Receptor-mediated antigen uptake and its effect on antigen presentation to class II-restricted T lymphocytes. Annu. Rev. Immunol..

[30] Mix E., Meyer-Rienecker H., Hartung H.P., Zettl U.K. (2010). Animal models of multiple sclerosis--potentials and limitations. Prog. Neurobiol..

[31] Mosmann T.R., Li L., Sad S. (1997). Functions of CD8 T-cell subsets secreting different cytokine patterns. Semin. Immunol..

[32] Damoiseaux J. (2006). Regulatory T cells: Back to the future. Neth. J. Med..

[33] Giardino L., Savoldi E., Pontieri F., Berutti E. (2004). Russell bodies in dental pulp of permanent human teeth. Oral Surg. Oral Med. Oral Pathol. Oral Radiol. Endod..

[34] Izumi T., Kobayashi I., Okamura K., Sakai H. (1995). Immunohistochemical study on the immunocompetent cells of the pulp in human non-carious and carious teeth. Arch. Oral. Biol..

[35] Bednarczyk M., Medina-Montano C., Fittler F.J., Stege H., Roskamp M., Kuske M., Langer C., Vahldieck M., Montermann E., Tubbe I. (2021). Complement-Opsonized Nano-Carriers Are Bound by Dendritic Cells (DC) via Complement Receptor (CR)3, and by B Cell Subpopulations via CR-1/2, and Affect the Activation of DC and B-1 Cells. Int. J. Mol. Sci..

[36] Lund F.E., Garvy B.A., Randall T.D., Harris D.P. (2005). Regulatory roles for cytokine-producing B cells in infection and autoimmune disease. Curr. Dir. Autoimmun..

[37] Hahn C.L., Falkler W.A., Siegel M.A. (1989). A study of T and B cells in pulpal pathosis. J. Endod..

[38] Speer M.L., Madonia J.V., Heuer M.A. (1977). Quantitative evaluation of the immunocompetence of the dental pulp. J. Endod..

[39] Wu S., Zhang L., Deng J., Guo B., Li F., Wang Y., Wu R., Zhang S., Lu J., Zhou Y. (2020). A Novel Micropeptide Encoded by Y-Linked LINC00278 Links Cigarette Smoking and AR Signaling in Male Esophageal Squamous Cell Carcinoma. Cancer Res..

[40] Yang C., Cao H., Yang J.W., Wang J.T., Yu M.M., Wang B.S. (2022). The ETS1-LINC00278 negative feedback loop plays a role in COL4A1/COL4A2 regulation in laryngeal squamous cell carcinoma. Neoplasma.

[41] Chen M., Zheng Y., Xie J., Zhen E., Zhou X. (2020). Integrative profiling analysis identifies the oncogenic long noncoding RNA DUXAP8 in oral cancer. Anticancer Drugs.

[42] Wang Y., Wang X., Xu Q., Yin J., Wang H., Zhang L. (2022). CircRNA, lncRNA, and mRNA profiles of umbilical cord blood exosomes from preterm newborns showing bronchopulmonary dysplasia. Eur. J. Pediatr..

[43] Chen G.R., Sun W., Zheng K., Zhu W. (2020). LINC01857 promotes the development of gastric cancer by regulating microRNA-200b. Eur. Rev. Med. Pharmacol. Sci..

[44] Li Q., Li B., Lu C.L., Wang J.Y., Gao M., Gao W. (2021). LncRNA LINC01857 promotes cell growth and diminishes apoptosis via PI3K/mTOR pathway and EMT process by regulating miR-141-3p/MAP4K4 axis in diffuse large B-cell lymphoma. Cancer Gene Ther..

[45] Hu G., Liu N., Wang H., Wang Y., Guo Z. (2019). LncRNA LINC01857 promotes growth, migration, and invasion of glioma by modulating miR-1281/TRIM65 axis. J. Cell Physiol..

[46] Liu L., Wang T., Huang D., Song D. (2021). Comprehensive Analysis of Differentially Expressed Genes in Clinically Diagnosed Irreversible Pulpitis by Multiplatform Data Integration Using a Robust Rank Aggregation Approach. J. Endod..

