# Supplementary material for: Identification and Experimental Validation of LINC00582 Associated with B Cell Immune and Development of Pulpitis: Bioinformatics and In Vitro Analysis
Source: Diagnostics (Basel). 2023 May 9;13(10):1678. doi: 10.3390/diagnostics13101678 (PMC10217272; doi:10.3390/diagnostics13101678)
Supplement: Supplementary file 1 [file diagnostics-13-01678-s001.zip › diagnostics-2279951-supplementary.pdf]

diagnostics

Supplementary Materials: Identification and Experimental Validation of LINC00582 Associated with B Cell Immune and Development of Pulpitis: Bioinformatics and In Vitro Analysis

Wenting Gong [1,2], Lilin Hong [1,2,*] and Yi Qian [1,2]

Table S1. The primers used in this study.

Gene name	Forward primer （5′-3′）	Reverse primer （5′-3′）
LINC02618	TGACTCCCCACCAAGAATCC	GCAGATCTCTTTCGCCAGTG
LINC02828	CTAAGGATGGAGATGGGGCC	CTCCAAGTTCCACCAGCTCT
LINC02482	CGGAATTGGTGGGTTCTTGG	TGAAGGTCCTGAGGCTGTTT
LINC00582	AGGCGGAAAGAACTACGTGA	TCACACCCAGACTCTGCATT
LINC01926	CTGAAAGCCATCCTCTTCGG	TCTCAGCCCTCTACAATGCC
LINC01094	AGCCTCGGCTGTGTTTGTAT	AGGTTGACACATCTCGCCTG
LINC02705	TTGGTGTGCCCTGAAGAGAT	GGCGTTCACATCATCTCCAC
LINC01266	TTGCTGCACCTCTCAACCTA	ACACAGGGAGGGAACAACAA
LINC00278	CAGGACAACTTCAACCCGAG	GAGGTCCTGTAGCACACTGT
LINC01724	CCTGCCCAGAGACTGTACAT	AAACCTGGGCCAAGTTAACT
LINC02561	GAGGGCATCAAAGAAGTGGA	TCCAGGGCTCAATCTCTGAC
LINC01857	CTCCACTGCGCTTTGTCCAT	GAGGCTTTGAGGATGGGGAC
ANKRD44-IT1	AAGGTGACATGAGGGGACAG	AAACACCCCTCGATTGCTTG
IL10RB-DT	AGGCCACCAAGAAGAGCTAG	CATTGGAGAGCGTACGTGTG
LINC02148	GGTGGGTATGGGAGACAGAG	AATCTGGTTCTCTCCTGGGC
LINC02123	CAGAGAACTTTGGCTGCACA	GCCTCCCCAGATTCCAAGTA
LINC01665	TGAAGCTCTGCCTCCTGAAG	TTCATTCTGCAGCCAAACCC
IL-6	ATGCTTCCAATCTGGATTCAATG	CAGCTCTGGCTTGTTCCTCACT
CD79B	CAGAGCCCACGTTTCATAGC	TCTCGTCCATCTCCTGCTTC
IL-8	CCAGGAAGAAACCACCGGA	GAAATCAGGAAGGCTGCCAAG
GAPDH	CCAAGGAGTAAGACCCCTGG	AGGGGAGATTCAGTGTGGTG
